# Supplementary material for: Prevalence and determinants of anxiety, depression, and suicidal ideation among adolescents of Parbat District: A cross-sectional study
Source: PLOS Ment Health. 2026 May 14;3(5):e0000613. doi: 10.1371/journal.pmen.0000613 (PMC13175331; doi:10.1371/journal.pmen.0000613)
Supplement: S1 Data — (PDF) [file pmen.0000613.s001.pdf]

SN	Class	Ageinyears	Sex	ethnew	MaritalSta	Religion	TypeofFam	LivingArran	MaritalStat	Numberof	FathersEdu	FathersEm	MothersEd	MothersEm	House_inc	Anydisabili	Anyfamily	Academic	SchoolAtte	Participati	SocialSupp	smokinghis	Alcoholhis	Drugabuse	Historyofm	Familyhist	HistoryofC	Accesstom	SBQR_CUT	PHQ_CUT	gad_cut_1
1.00	3	17	0	4.00	0	0	0	0	1	1	3	2	3	0	1.00	0	0	1	1	0	0	1	0	0	0	0	0	0	2.00	1.00	1.00
2.00	3	18	1	4.00	0	0	1	3	1	0	3	1	2	0	1.00	0	0	1	1	1	0	0	0	0	0	0	0	0	1.00	1.00	1.00
3.00	3	18	1	4.00	0	0	0	1	1	1	2	2	2	0	2.00	0	0	0	1	1	1	0	0	0	0	0	0	0	1.00	1.00	2.00
4.00	3	18	1	4.00	0	0	0	1	1	2	2	2	2	0	1.00	0	0	1	1	1	1	0	0	0	0	0	0	0	1.00	2.00	1.00
5.00	3	16	1	1.00	0	0	0	1	1	1	3	2	3	1	2.00	0	0	2	1	1	1	0	0	0	0	0	0	0	1.00	1.00	1.00
6.00	3	17	1	1.00	0	0	0	1	1	2	3	1	3	0	1.00	0	0	1	1	1	1	0	0	0	1	0	0	0	1.00	1.00	1.00
7.00	3	18	1	1.00	0	0	1	2	1	1	3	2	3	0	2.00	0	0	1	1	1	1	0	0	0	0	0	0	0	1.00	1.00	1.00
8.00	1	14	1	4.00	0	0	0	0	1	1	3	2	3	0	1.00	0	0	0	1	1	0	0	1	0	0	0	0	0	1.00	1.00	1.00
9.00	3	17	1	4.00	0	0	1	1	1	0	2	1	2	1	1.00	0	0	1	1	1	0	0	0	0	0	0	0	0	1.00	1.00	1.00
10.00	3	16	1	1.00	0	1	1	2	1	1	2	1	2	1	1.00	0	0	2	1	1	1	0	0	0	0	0	0	0	1.00	1.00	1.00
11.00	3	18	1	4.00	0	0	0	1	1	1	3	3	3	0	2.00	0	0	0	1	1	1	1	0	0	0	0	1	1	1.00	1.00	2.00
12.00	3	17	1	4.00	0	0	1	2	0	1	2	2	3	0	1.00	0	0	1	1	1	0	0	0	0	0	0	1	1	1.00	2.00	2.00
13.00	3	17	1	0.00	0	0	1	2	1	2	2	2	3	2	1.00	0	0	1	1	1	0	0	0	0	0	0	0	0	1.00	1.00	1.00
14.00	3	18	1	1.00	0	0	0	1	1	2	2	2	2	1	2.00	0	0	0	1	1	0	0	0	0	0	0	0	0	1.00	1.00	2.00
15.00	3	16	1	4.00	0	0	0	0	0	0	2	2	2	1	2.00	0	1	0	1	1	0	0	0	0	1	0	0	0	1.00	1.00	2.00
16.00	3	18	1	1.00	0	0	1	2	1	0	3	0	2	0	1.00	1	1	1	1	1	1	0	0	0	0	0	0	0	1.00	1.00	2.00
17.00	3	19	1	1.00	0	1	1	0	1	0	2	2	2	0	2.00	0	0	0	1	1	1	1	1	0	0	0	1	1	1.00	1.00	2.00
18.00	3	17	1	4.00	0	0	0	0	1	0	3	2	3	2	2.00	0	0	0	1	1	1	0	0	0	0	0	0	0	1.00	1.00	2.00
19.00	3	17	1	4.00	0	0	1	1	1	0	3	1	3	3	1.00	0	0	1	1	1	1	0	0	0	0	0	0	0	1.00	1.00	2.00
20.00	3	17	1	4.00	0	0	0	1	1	1	3	2	3	1	2.00	0	0	1	1	1	0	0	0	0	0	0	0	0	1.00	1.00	2.00
21.00	3	18	1	4.00	0	0	1	2	1	2	3	2	3	3	2.00	0	0	1	1	1	0	0	0	0	0	0	0	0	1.00	1.00	2.00
22.00	3	16	1	0.00	0	0	0	0	1	0	3	1	3	3	2.00	0	0	1	1	1	1	0	0	0	0	0	0	0	1.00	1.00	2.00
23.00	3	17	1	4.00	0	0	0	0	0	0	2	1	2	0	2.00	0	0	0	1	1	0	0	0	0	0	0	0	0	1.00	1.00	2.00
24.00	3	17	1	1.00	0	1	0	0	1	2	3	1	3	1	2.00	0	0	0	1	1	1	0	0	0	0	0	0	0	1.00	1.00	2.00
25.00	3	17	1	4.00	0	0	0	1	1	2	3	0	3	1	1.00	0	0	1	1	1	0	0	0	0	0	0	0	0	1.00	1.00	2.00
26.00	3	17	1	1.00	0	0	0	1	1	1	2	2	2	2	2.00	0	0	0	1	1	0	0	0	0	0	0	0	0	1.00	1.00	2.00
27.00	3	16	1	1.00	0	1	0	0	1	1	2	2	2	0	1.00	0	0	1	1	1	0	0	0	0	0	0	0	0	1.00	1.00	2.00
28.00	3	18	1	0.00	0	0	0	0	1	2	2	2	2	1	1.00	0	0	1	1	0	1	0	0	0	0	0	0	0	1.00	1.00	2.00
29.00	3	18	1	4.00	0	0	1	0	1	0	3	2	3	0	2.00	0	0	1	1	1	1	0	0	0	0	0	0	0	1.00	1.00	2.00
30.00	3	17	1	1.00	0	1	1	1	1	1	3	1	2	1	2.00	0	0	1	1	1	0	0	0	0	0	0	0	0	1.00	1.00	2.00
31.00	3	17	1	4.00	0	0	0	0	0	0	2	2	2	0	1.00	0	1	0	1	1	0	0	0	0	0	0	1	0	1.00	1.00	2.00
32.00	3	16	1	4.00	0	0	0	2	1	0	2	2	3	1	1.00	0	0	0	1	1	0	0	0	0	0	0	0	0	1.00	1.00	2.00
33.00	3	17	1	0.00	0	0	1	2	1	1	3	3	3	3	1.00	0	0	0	1	0	1	0	0	0	0	0	0	0	1.00	1.00	2.00
34.00	3	16	1	4.00	0	0	0	0	1	0	2	1	2	1	1.00	0	0	1	1	1	0	0	0	0	0	0	0	0	1.00	1.00	2.00
35.00	1	15	1	1.00	0	1	1	2	1	0	2	2	2	1	1.00	0	0	1	1	0	0	0	0	0	0	0	0	0	1.00	1.00	2.00
36.00	3	18	1	1.00	0	1	1	0	0	0	3	1	3	0	2.00	0	0	1	1	1	1	0	0	0	0	0	0	0	1.00	1.00	2.00
37.00	1	15	1	1.00	0	0	1	2	1	1	3	1	3	0	1.00	0	0	0	1	1	0	0	0	0	0	0	0	0	1.00	2.00	2.00
38.00	1	15	1	1.00	0	1	1	1	1	0	3	1	3	0	2.00	0	0	1	1	0	0	0	0	0	0	0	0	0	1.00	2.00	2.00
39.00	3	16	1	1.00	0	0	1	2	1	0	2	1	2	1	1.00	0	0	1	1	1	0	0	0	0	0	0	1	0	1.00	2.00	2.00
40.00	3	16	1	0.00	0	0	1	2	1	0	3	1	3	1	2.00	0	0	1	1	1	0	0	0	0	0	0	0	0	1.00	2.00	2.00
41.00	3	17	1	4.00	0	1	0	0	1	0	3	2	2	0	1.00	0	0	0	1	1	0	0	0	0	0	0	0	0	1.00	2.00	2.00
42.00	3	17	1	4.00	0	0	0	1	1	1	2	2	2	1	1.00	0	0	1	1	1	0	0	0	0	0	0	0	0	1.00	2.00	2.00
43.00	3	16	1	4.00	0	0	1	0	1	0	3	2	2	0	1.00	0	0	0	1	1	1	0	0	0	0	0	0	0	1.00	2.00	2.00
44.00	3	17	1	0.00	0	1	0	1	1	0	2	3	2	1	2.00	0	0	1	1	1	0	0	0	0	0	0	0	0	1.00	2.00	2.00
45.00	1	15	1	1.00	0	0	0	0	0	0	2	1	3	0	2.00	0	0	1	0	1	1	0	0	0	0	0	0	0	1.00	2.00	2.00
46.00	3	16	1	1.00	0	1	0	1	1	1	2	3	2	1	2.00	0	0	0	1	1	0	0	0	0	0	0	0	0	1.00	2.00	2.00
47.00	3	16	1	1.00	0	0	1	2	1	1	2	1	2	0	1.00	1	0	0	1	1	0	0	0	0	1	0	0	0	1.00	2.00	2.00
48.00	3	18	1	4.00	0	0	0	0	1	0	3	2	3	2	1.00	0	0	1	1	1	0	0	0	0	0	0	0	0	1.00	2.00	2.00
49.00	3	16	1	4.00	0	0	1	0	1	1	3	0	3	0	1.00	0	0	0	1	0	1	0	0	0	0	0	0	0	1.00	2.00	2.00
50.00	1	15	1	1.00	0	1	0	0	1	0	3	1	3	1	2.00	0	0	1	1	1	0	0	0	0	0	0	0	0	1.00	2.00	2.00
51.00	3	17	1	1.00	0	1	1	0	1	1	3	2	3	0	1.00	0	0	1	1	0	1	0	0	0	0	0	1	0	1.00	2.00	2.00
52.00	3	16	1	4.00	0	0	1	1	1	1	2	1	2	1	1.00	0	0	1	1	1	0	0	0	0	0	1	0	0	1.00	2.00	2.00
53.00	3	16	1	4.00	0	0	0	0	1	0	3	2	3	0	2.00	0	0	0	1	1	0	0	0	0	0	0	0	0	1.00	2.00	2.00
54.00	3	16	1	4.00	0	0	0	1	1	1	2	2	2	1	1.00	0	0	0	1	1	0	0	0	0	0	0	1	1	1.00	2.00	2.00
55.00	3	17	1	4.00	0	0	0	3	1	0	3	1	3	1	1.00	0	0	0	1	1	0	0	0	0	0	0	0	0	1.00	2.00	2.00
56.00	3	17	1	0.00	0	0	0	1	1	0	3	3	3	0	2.00	1	0	3	1	0	1	0	1	0	0	0	1	0	1.00	2.00	2.00
57.00	3	17	1	1.00	0	0	1	2	1	0	2	1	2	1	1.00	0	0	1	1	0	1	0	0	0	0	0	0	0	1.00	2.00	2.00
58.00	3	16	1	4.00	0	0	1	3	1	1	2	1	2	1	2.00	0	0	1	1	0	1	0	0	0	0	0	0	0	1.00	2.00	2.00
59.00	3	18	1	4.00	0	0	0	3	1	1	3	1	3	0	2.00	0	0	1	1	1	1	0	0	0	0	0	0	0	1.00	2.00	2.00
60.00	3	18	1	4.00	0	0	0	3	1	1	3	1	3	1	1.00	0	0	1	1	1	1	0	0	0	0	0	0	0	1.00	2.00	2.00
61.00	3	18	1	4.00	0	0	0	3	1	1	3	3	3	1	2.00	0	0	0	1	1	1	0	0	0	0	0	0	0	1.00	2.00	2.00
62.00	3	17	0	4.00	0	0	0	1	1	2	2	1	3	1	2.00	0	0	1	1	1	1	0	0	0	0	0	0	0	1.00	2.00	2.00
63.00	3	17	0	4.00	0	0	0	0	1	0	2	0	2	0	1.00	0	0	0	1	1	0	0	0	0	0	0	0	0	1.00	2.00	2.00
64.00	1	15	1	4.00	0	0	0	2	1	1	2	2	2	0	1.00	0	0	0	1	1	0	0	0	0	0	0	0	0	1.00	2.00	2.00
65.00	3	17	1	4.00	0	0	0	3	1	1	2	2	3	0	2.00	0	0	1	1	1	0	0	0	0	0	0	0	0	1.00	1.00	2.00
66.00	3	20	0	1.00	0	0	0	3	1	2	3	1	2	0	1.00	0	0	2	1	1	1	0	0	0	1	0	0	1	1.00	1.00	2.00
67.00	3	17	0	0.00	0	1	0	1	1	0	3	1	2	1	1.00	0	0	0	1	1	0	0	0	0	0	0	0	0	1.00	2.00	2.00
68.00	3	20	0	1.00	0	0	0	3	1	2	3	2	3	0	1.00	0	0	0	1	1	1	0	0	0	0	0	0	0	1.00	1.00	2.00
69.00	3	18	1	0.00	0	0	0	0	0	0	3	1	3	1	1.00	0	0	2	1	0	1	0	0	0	0	0	0	0	1.00	1.00	2.00
70.00	3	16	0	4.00	0	0	0	1	1	1	3	2	3	1	2.00	0	0	1	1	1	0	0	0	0	0	0	0	0	1.00	1.00	2.00
71.00	3	17	0	0.00	0	1	0	1	1	2	3	1	3	1	1.00	0	0	2	1	1	1	0	0	0	0	0	0	0	1.00	1.00	2.00
72.00	3	16	0	0.00	0	0	0	1	1	2	2	1	2	0	1.00	0	0	1	1	1	1	0	0	0	0	0	0	0	1.00	1.00	2.00
73.00	3	17	0	4.00	0	0	0	3	1	1	2	1	2	0	1.00	0	0	0	1	1	1	0	0	0	0	0	0	0	1.00	1.00	2.00
74.00	3	16	1	4.00	0	0	1	2	1	2	2	1	3	1	1.00	0	0	1	1	1	0	0	0	0	0	0	0	0	1.00	1.00	2.00
75.00	3	16	1	4.00	0	0	1	1	1	2	3	1	3	0	1.00	0	0	1	1	1	1	0	0	0	0	0	0	0	1.00	1.00	2.00
76.00	3	17	1	0.00	0	0	1	1	1	1	3	3	3	3	2.00	0	0	1	1	0	1	0	0	0	0	0	0	0	1.00	1.00	2.00
77.00	3	16	0	4.00	0	0	0	3	1	0	3	3	2	0	2.00	0	0	0	1	1	1	0	0	0	0	0	0	0	1.00	1.00	2.00
78.00	3	18	0	0.00	0	1	1	1	0	2	2	1	3	1	1.00	0	0	0	1	0	1	1	1	1	0	0	0	0	1.00	1.00	2.00
79.00	3	19	0	4.00	0	0	0	1	1	1	3	1	2	1	1.00	0	0	1	1	1	1	0	0	0	0	0	0	0	1.00	1.00	2.00
80.00	3	16	0	4.00	0	0	1	1	1	1	2	1	2	1	2.00	0	0	1	1	1	0	0	0	0	0	0	0	0	1.00	1.00	2.00
81.00	3	17	0	4.00	0	0	0	1	1	1	3	3	3	0	2.00	0	0	0	1	1	1	0	0	0	0	0	0	0	1.00	1.00	2.00

82.00	3	18	0	4.00	0	0	0	1	1	1	3	1	3	1	2.00	0	0	2	1	0	1	0	0	0	0	0	0	0	1.00	1.00	2.00
83.00	3	18	1	0.00	0	0	1	2	0	2	2	2	3	0	1.00	0	0	1	1	1	0	0	0	0	0	0	0	0	1.00	1.00	2.00
84.00	3	19	1	0.00	0	0	1	1	1	2	3	1	3	1	1.00	0	0	3	1	0	1	0	0	0	0	0	0	0	1.00	1.00	2.00
85.00	3	17	1	4.00	0	0	0	0	1	2	2	1	2	1	1.00	0	0	2	1	1	0	0	0	0	0	0	0	0	1.00	1.00	2.00
86.00	3	17	0	4.00	0	0	0	3	1	0	3	3	3	1	1.00	0	0	0	1	1	1	0	0	0	0	0	0	0	1.00	1.00	2.00
87.00	3	17	1	0.00	0	0	0	0	1	1	2	1	2	1	2.00	0	0	1	1	1	1	0	0	0	0	0	0	0	1.00	1.00	2.00
88.00	3	18	1	0.00	0	0	1	2	1	1	2	1	2	1	1.00	0	0	0	1	1	1	0	0	0	0	0	0	0	1.00	1.00	2.00
89.00	3	19	1	4.00	0	0	0	3	1	1	2	1	2	0	1.00	0	0	0	1	1	0	0	0	0	0	0	0	0	1.00	1.00	2.00
90.00	3	18	0	4.00	0	0	0	1	1	1	2	1	2	1	1.00	0	0	1	1	1	1	0	0	0	0	0	0	0	1.00	1.00	2.00
91.00	3	17	1	0.00	0	0	1	1	1	2	3	1	2	1	2.00	0	0	1	1	1	0	0	0	0	0	0	0	0	1.00	1.00	2.00
92.00	3	16	1	4.00	0	0	1	3	1	1	3	2	3	0	2.00	0	0	1	1	1	0	0	0	0	0	0	0	0	1.00	1.00	2.00
93.00	3	19	1	0.00	0	0	1	2	1	2	3	1	2	1	1.00	0	0	1	1	1	0	0	0	0	0	0	0	0	1.00	1.00	2.00
94.00	3	17	0	4.00	0	0	0	0	1	1	3	3	3	0	1.00	0	0	1	1	1	1	0	0	0	0	0	0	0	1.00	1.00	2.00
95.00	3	19	1	0.00	1	0	1	3	1	2	3	1	3	0	1.00	1	0	2	0	1	0	0	0	0	0	0	0	0	1.00	1.00	2.00
96.00	3	18	0	4.00	0	0	0	1	1	2	2	3	2	0	1.00	1	0	1	1	1	1	0	0	0	0	0	0	0	1.00	1.00	2.00
97.00	3	16	0	4.00	0	0	0	3	1	2	3	3	3	0	2.00	0	0	1	1	1	1	0	0	0	0	0	0	0	1.00	1.00	2.00
98.00	3	18	1	4.00	0	0	0	1	1	1	2	1	2	1	1.00	0	0	2	1	1	0	0	0	0	0	0	0	0	1.00	1.00	1.00
99.00	3	16	0	4.00	0	0	0	3	1	2	3	1	3	1	2.00	0	0	1	1	1	1	0	0	0	0	0	0	0	1.00	1.00	1.00
100.00	3	18	1	1.00	0	1	1	1	1	2	2	1	2	1	1.00	0	0	3	0	0	1	1	0	0	0	0	1	1	1.00	2.00	1.00
101.00	3	16	1	4.00	0	0	0	0	1	0	3	1	3	0	2.00	0	1	0	1	1	0	0	0	0	0	0	0	0	1.00	1.00	2.00
102.00	3	16	0	4.00	0	0	0	1	1	1	3	1	3	0	1.00	0	0	1	1	1	1	0	0	0	1	0	0	0	1.00	1.00	1.00
103.00	3	16	0	4.00	0	0	0	0	1	1	2	1	2	0	1.00	0	0	0	1	1	1	0	0	0	0	0	0	0	1.00	1.00	1.00
104.00	3	17	1	4.00	0	0	1	2	1	0	3	2	3	2	2.00	0	0	2	1	0	0	0	0	0	0	0	0	0	1.00	1.00	1.00
105.00	3	17	1	1.00	0	0	0	1	1	2	3	2	3	0	1.00	0	0	1	1	1	1	0	0	0	0	0	0	0	1.00	1.00	1.00
106.00	3	17	1	4.00	0	0	0	1	1	0	3	2	3	1	2.00	0	0	0	1	1	0	0	0	0	0	0	0	0	1.00	1.00	1.00
107.00	3	17	0	1.00	0	0	0	3	1	1	2	2	2	0	1.00	0	0	1	1	1	1	0	0	0	0	0	0	0	1.00	1.00	1.00
108.00	3	17	1	1.00	0	0	0	3	1	1	2	1	2	1	2.00	0	0	2	0	0	0	0	0	0	0	0	0	0	1.00	1.00	1.00
109.00	3	17	1	1.00	0	0	0	3	1	0	2	0	2	0	1.00	0	0	0	1	0	0	0	0	0	1	0	1	0	1.00	2.00	2.00
110.00	3	17	0	4.00	0	0	0	3	1	1	3	1	3	0	2.00	0	0	1	1	0	0	1	0	0	0	0	0	0	1.00	1.00	1.00
111.00	3	17	1	4.00	0	0	0	3	1	0	2	1	2	1	2.00	0	0	3	1	1	0	0	0	0	0	0	0	0	1.00	1.00	1.00
112.00	3	18	1	1.00	0	1	1	2	1	0	2	1	2	0	1.00	0	0	2	1	1	0	0	0	0	0	0	0	0	1.00	1.00	1.00
113.00	3	18	0	4.00	0	0	0	0	1	1	2	1	2	1	1.00	1	1	2	1	0	0	0	0	0	0	0	0	0	1.00	1.00	1.00
114.00	3	18	0	4.00	0	0	0	3	1	1	2	1	2	1	1.00	1	0	1	1	1	0	0	0	0	0	0	1	0	2.00	1.00	1.00
115.00	3	17	0	0.00	0	0	0	1	1	2	3	1	3	3	1.00	0	0	0	1	1	1	0	0	0	0	0	0	0	1.00	1.00	1.00
116.00	3	17	1	4.00	0	0	1	3	1	2	3	2	3	1	2.00	0	0	1	1	1	0	0	0	0	0	0	0	0	1.00	1.00	1.00
117.00	3	17	1	1.00	0	0	1	2	1	1	3	2	3	1	2.00	0	0	2	1	1	0	0	0	0	0	0	0	0	1.00	1.00	1.00
118.00	3	17	1	1.00	0	0	0	3	1	1	3	3	3	0	2.00	0	0	1	1	1	1	0	0	0	0	0	0	0	1.00	1.00	1.00
119.00	3	17	1	1.00	0	0	1	3	1	1	3	1	3	1	1.00	0	0	1	1	1	1	0	0	0	0	0	0	0	1.00	1.00	1.00
120.00	3	16	1	1.00	0	0	0	0	1	2	3	1	3	0	1.00	0	0	2	1	1	0	0	0	0	0	0	0	0	1.00	1.00	1.00
121.00	3	17	0	0.00	0	0	1	3	1	2	2	2	2	0	1.00	0	0	1	0	0	1	0	0	0	0	0	0	0	1.00	1.00	1.00
122.00	3	17	1	1.00	0	0	0	0	1	1	3	1	2	1	1.00	0	0	1	1	0	1	0	0	0	0	0	0	0	1.00	1.00	1.00
123.00	3	18	1	0.00	0	0	0	1	1	2	3	2	3	1	2.00	0	0	2	1	0	0	0	0	0	0	0	1	0	2.00	2.00	2.00
124.00	3	16	1	4.00	0	0	0	3	1	1	2	1	2	1	1.00	0	0	1	1	1	0	0	0	0	0	0	0	0	1.00	1.00	1.00
125.00	3	19	0	4.00	0	0	0	0	1	1	3	0	3	1	1.00	0	0	1	1	1	1	0	0	0	0	0	0	0	1.00	1.00	1.00
126.00	3	17	1	4.00	0	0	0	3	1	2	3	1	2	1	1.00	0	1	1	1	0	1	0	0	0	0	0	0	0	1.00	1.00	1.00
127.00	3	17	1	0.00	0	0	1	2	1	2	2	1	3	1	1.00	0	0	2	1	0	0	0	0	0	0	0	0	0	1.00	1.00	1.00
128.00	3	17	1	0.00	0	0	0	1	1	2	3	1	2	0	1.00	0	0	1	1	0	0	0	0	0	0	0	0	0	1.00	1.00	1.00
129.00	3	16	0	1.00	0	0	0	3	1	1	3	1	3	1	2.00	0	0	1	1	1	1	0	0	0	1	0	0	1	1.00	1.00	1.00
130.00	3	18	0	1.00	0	0	0	0	1	2	2	2	2	3	1.00	0	0	1	1	1	0	0	0	0	0	0	0	0	1.00	1.00	1.00
131.00	3	17	1	4.00	0	0	1	2	1	2	3	2	3	3	2.00	0	0	0	1	1	0	0	0	0	0	0	0	0	1.00	1.00	1.00
132.00	3	17	0	4.00	0	0	0	0	1	0	3	1	2	0	2.00	0	0	0	1	0	0	0	0	0	0	0	0	0	1.00	1.00	1.00
133.00	3	17	0	4.00	0	0	0	0	1	0	3	0	3	1	1.00	0	0	1	1	0	0	0	0	0	0	0	0	0	2.00	1.00	1.00
134.00	3	17	0	0.00	0	0	0	0	1	1	2	3	2	3	2.00	0	0	1	0	1	0	0	0	0	0	0	0	0	1.00	1.00	1.00
135.00	3	16	1	4.00	0	0	0	1	1	1	2	1	2	1	1.00	0	0	0	1	1	0	0	0	0	0	0	0	0	1.00	1.00	1.00
136.00	3	17	0	0.00	0	1	0	1	1	0	3	3	3	1	2.00	0	0	1	1	0	0	0	0	0	0	0	0	0	1.00	1.00	1.00
137.00	3	16	0	4.00	0	0	0	1	1	0	3	0	2	1	2.00	0	0	1	1	1	1	0	0	0	0	0	0	0	1.00	1.00	1.00
138.00	3	18	1	1.00	0	0	0	0	1	0	3	1	2	0	1.00	0	0	1	1	1	0	0	0	0	0	0	0	0	1.00	1.00	1.00
139.00	3	17	1	4.00	0	0	0	3	1	1	2	1	3	1	2.00	0	0	0	1	1	0	0	0	0	0	0	0	0	1.00	1.00	1.00
140.00	3	19	1	1.00	0	0	0	0	0	0	3	2	3	3	2.00	0	0	1	1	1	0	0	0	0	0	0	0	0	1.00	1.00	1.00
141.00	3	17	1	4.00	0	0	0	1	1	0	3	1	2	1	1.00	0	0	1	1	1	0	0	0	0	0	0	0	0	1.00	1.00	1.00
142.00	3	17	0	4.00	0	0	0	0	1	1	2	2	3	1	2.00	0	0	1	1	1	0	0	0	0	0	0	0	0	1.00	1.00	1.00
143.00	3	18	1	4.00	0	0	1	2	1	2	3	2	3	2	2.00	0	0	1	1	1	0	0	0	0	0	0	0	0	1.00	1.00	1.00
144.00	3	18	0	1.00	0	1	1	3	0	1	2	1	2	2	2.00	0	0	2	1	1	0	0	0	0	0	0	0	0	1.00	1.00	1.00
145.00	3	17	1	1.00	0	1	0	1	1	1	2	1	2	1	2.00	0	0	2	1	1	0	0	0	0	1	0	0	0	2.00	2.00	2.00
146.00	3	16	1	1.00	0	1	0	1	1	1	3	1	3	0	1.00	0	0	2	1	1	0	0	0	0	1	0	0	0	2.00	2.00	2.00
147.00	3	17	0	4.00	0	0	1	2	1	1	3	1	3	1	1.00	1	0	2	0	1	1	1	1	1	0	0	0	0	1.00	1.00	1.00
148.00	3	18	1	4.00	0	0	0	1	1	1	3	2	3	1	2.00	0	0	2	1	1	0	0	0	0	0	0	0	0	1.00	1.00	1.00
149.00	3	18	1	1.00	0	0	0	0	0	0	3	1	2	0	1.00	0	0	2	1	1	0	0	0	0	0	0	0	0	1.00	1.00	1.00
150.00	3	16	0	1.00	0	0	1	1	1	0	3	0	3	0	1.00	0	0	2	1	1	0	0	0	0	0	0	0	0	1.00	1.00	1.00
151.00	3	18	0	4.00	0	0	0	3	1	1	2	2	2	1	2.00	0	0	1	1	1	0	0	0	0	0	0	0	0	1.00	1.00	1.00
152.00	3	17	0	4.00	0	0	0	3	1	2	2	2	2	1	2.00	0	0	1	1	1	0	0	0	0	0	0	0	0	1.00	1.00	1.00
153.00	3	17	1	4.00	0	0	1	2	1	2	2	1	2	0	1.00	0	0	1	1	1	0	0	0	0	0	0	0	0	1.00	1.00	1.00
154.00	3	18	1	1.00	0	0	0	3	1	1	2	1	2	1	1.00	0	0	1	1	0	0	0	0	0	0	0	0	0	1.00	1.00	1.00
155.00	3	17	1	1.00	0	0	0	1	1	1	3	1	2	1	1.00	0	0	1	1	0	0	0	1	0	0	0	0	0	1.00	1.00	1.00
156.00	3	17	0	4.00	0	0	0	2	1	0	2	0	2	1	1.00	1	0	2	0	0	0	0	0	0	1	0	0	1	1.00	1.00	2.00
157.00	3	17	1	4.00	0	0	0	1	1	1	2	1	3	1	1.00	0	0	1	1	0	0	0	0	0	0	0	0	0	1.00	1.00	1.00
158.00	3	17	1	1.00	0	0	0	1	1	0	3	2	3	0	2.00	0	0	1	1	1	0	0	0	0	0	0	0	0	1.00	1.00	1.00
159.00	3	17	1	1.00	0	0	0	3	1	2	3	3	3	3	1.00	0	0	1	1	1	0	0	0	0	0	0	0	0	1.00	1.00	1.00
160.00	3	17	1	0.00	0	1	0	1	1	1	3	2	3	0	1.00	0	0	0	1	1	1	0	0	0	0	0	0	0	1.00	1.00	1.00
161.00	3	18	0	4.00	0	0	1	3	1	1	2	1	3	0	2.00	1	0	1	1	0	1	0	0	0	0	0	0	0	1.00	1.00	1.00
162.00	1	14	1	1.00	0	0	0	1	1	0	2	1	2	0	1.00	0	0	1	1	1	0	0	0	0	0	0	0	0	1.00	1.00	1.00
163.00	3	16	1	4.00	0	0	0	0	1	0	2	2	2	1	1.00	0	0	1	1	1	0	0	0	0	0	0	0	0	1.00	1.00	1.00

164.00	3	17	1	1.00	0	1	0	3	1	1	2	0	2	1	1.00	0	0	1	1	1	1	0	0	0	1	0	1	1	2.00	2.00	2.00
165.00	3	17	0	1.00	0	0	1	3	1	1	2	2	3	2	2.00	0	1	2	1	1	0	0	0	0	0	0	0	0	1.00	1.00	1.00
166.00	3	17	1	4.00	0	0	0	0	1	1	2	0	2	0	1.00	0	0	1	1	1	0	0	0	0	0	0	0	0	1.00	1.00	1.00
167.00	3	17	1	1.00	0	0	1	2	1	1	2	1	2	1	1.00	0	0	0	1	1	1	0	0	0	0	0	0	0	1.00	1.00	1.00
168.00	3	18	0	4.00	0	0	0	1	1	0	2	2	2	1	1.00	0	1	1	1	0	0	0	0	0	0	0	0	0	1.00	1.00	1.00
169.00	3	17	0	1.00	0	0	1	1	1	1	2	0	2	0	1.00	0	0	2	1	0	0	0	0	0	0	0	0	0	1.00	1.00	1.00
170.00	3	17	1	4.00	0	0	1	3	1	2	2	0	2	0	1.00	0	0	0	1	1	0	0	0	0	0	0	0	0	1.00	1.00	1.00
171.00	3	18	0	4.00	0	0	0	0	1	1	2	1	2	1	2.00	0	0	0	1	0	0	0	0	0	0	0	0	0	1.00	1.00	1.00
172.00	3	17	1	4.00	0	0	0	3	1	2	2	2	2	1	1.00	0	0	1	1	1	1	0	0	0	0	0	0	0	1.00	2.00	2.00
173.00	3	16	1	4.00	0	0	1	3	1	2	2	1	2	1	1.00	0	0	1	1	0	1	0	0	0	0	0	0	0	1.00	2.00	2.00
174.00	3	17	0	4.00	0	0	1	3	1	2	3	0	3	1	2.00	0	0	1	1	1	0	0	0	0	0	0	0	0	1.00	1.00	1.00
175.00	3	19	1	1.00	0	1	1	2	1	2	3	3	2	0	1.00	0	0	1	1	0	1	0	0	0	1	0	1	1	2.00	2.00	2.00
176.00	3	16	1	4.00	0	0	0	1	1	2	3	1	3	1	1.00	0	0	1	1	1	0	0	0	0	0	0	0	0	1.00	1.00	1.00
177.00	3	17	1	4.00	0	0	1	3	0	0	2	1	2	0	1.00	0	0	2	1	0	0	0	0	0	0	0	1	1	2.00	1.00	1.00
178.00	3	16	1	4.00	0	0	1	3	1	1	3	1	3	1	1.00	0	0	0	1	1	0	0	0	0	0	0	0	0	1.00	1.00	1.00
179.00	3	16	0	1.00	0	0	1	3	1	0	3	2	2	0	2.00	0	0	2	1	0	0	1	0	0	0	0	1	0	1.00	1.00	1.00
180.00	3	17	1	4.00	0	0	0	3	1	2	3	1	3	1	1.00	0	0	1	1	0	1	0	0	0	0	0	0	0	1.00	1.00	1.00
181.00	3	17	1	4.00	0	0	0	3	1	2	3	1	3	0	1.00	0	0	1	1	1	1	0	0	0	0	0	0	0	1.00	1.00	1.00
182.00	3	16	0	1.00	0	0	1	0	1	1	3	2	2	0	1.00	0	0	1	1	0	0	1	0	0	0	0	0	0	1.00	1.00	1.00
183.00	3	17	1	1.00	0	0	0	3	1	2	3	2	3	1	2.00	0	0	1	1	0	0	0	0	0	0	0	0	0	1.00	1.00	1.00
184.00	3	18	1	4.00	0	0	1	3	0	2	2	1	2	1	1.00	0	0	0	1	1	0	0	0	0	0	0	0	0	1.00	1.00	1.00
185.00	3	16	1	4.00	0	0	0	1	1	2	2	1	2	1	1.00	0	0	1	1	1	0	0	0	0	0	0	0	0	1.00	1.00	1.00
186.00	3	17	1	4.00	0	0	0	3	1	1	2	1	2	1	1.00	1	0	1	1	0	1	0	0	0	0	0	0	0	1.00	1.00	1.00
187.00	3	16	1	4.00	0	0	0	3	1	2	3	2	3	1	2.00	0	0	1	1	1	0	0	0	0	0	0	0	0	1.00	1.00	1.00
188.00	3	18	1	4.00	0	0	1	3	0	1	2	2	2	0	2.00	0	0	0	1	1	0	0	0	0	0	0	0	0	1.00	1.00	1.00
189.00	3	17	1	4.00	0	0	0	3	1	1	3	1	3	1	1.00	0	0	0	1	1	0	0	0	0	0	0	0	0	1.00	2.00	1.00
190.00	3	17	1	0.00	0	0	1	2	1	1	2	1	2	1	2.00	0	0	1	1	1	1	0	0	0	0	0	0	0	1.00	1.00	1.00
191.00	3	17	1	4.00	0	0	0	3	1	1	2	1	2	1	2.00	0	0	1	1	1	1	0	0	0	0	0	0	0	1.00	1.00	1.00
192.00	3	17	1	4.00	0	0	0	3	1	2	3	2	3	1	2.00	0	0	1	1	1	1	0	0	0	0	0	0	0	1.00	1.00	1.00
193.00	3	16	1	4.00	0	0	1	2	1	2	2	1	2	0	1.00	0	0	1	1	1	0	0	0	0	0	0	0	0	1.00	1.00	1.00
194.00	3	17	1	4.00	0	0	1	1	1	2	2	1	3	1	1.00	0	0	1	1	1	0	0	0	0	0	0	0	0	1.00	1.00	1.00
195.00	3	17	1	0.00	0	0	0	0	1	2	2	1	3	0	1.00	0	0	2	1	1	0	0	0	0	0	0	0	0	1.00	1.00	1.00
196.00	3	16	1	4.00	0	0	0	0	1	1	3	2	2	1	2.00	0	0	1	1	1	0	0	0	0	0	0	0	0	1.00	1.00	1.00
197.00	3	16	1	4.00	0	0	1	3	1	2	2	1	2	1	2.00	0	0	2	1	1	0	0	0	0	0	0	0	0	1.00	1.00	1.00
198.00	3	16	1	4.00	0	0	0	0	1	2	3	3	2	1	2.00	1	0	0	1	1	0	0	0	0	0	0	0	0	1.00	1.00	1.00
199.00	1	15	1	4.00	0	0	1	0	1	1	2	0	2	1	1.00	0	0	0	1	1	0	0	0	0	1	0	0	0	1.00	1.00	1.00
200.00	3	17	1	4.00	0	0	1	2	1	0	3	1	3	1	2.00	0	0	1	1	0	0	0	0	0	0	1	0	0	1.00	1.00	1.00
201.00	3	17	1	0.00	0	0	1	0	1	2	2	1	2	0	1.00	0	0	2	1	0	0	0	0	0	1	0	0	0	1.00	1.00	1.00
202.00	1	15	0	1.00	0	0	1	3	1	1	3	0	3	0	1.00	0	0	1	1	1	1	0	0	0	0	0	0	0	1.00	1.00	1.00
203.00	3	18	1	1.00	0	0	0	1	1	0	2	1	2	0	2.00	0	0	2	1	1	0	0	0	0	0	0	0	0	1.00	1.00	1.00
204.00	3	17	0	4.00	0	0	1	2	1	0	2	1	2	1	2.00	0	0	0	1	1	0	0	0	0	0	0	0	0	1.00	1.00	1.00
205.00	1	15	0	0.00	0	0	1	1	1	2	3	3	3	0	2.00	1	1	0	1	1	0	0	0	0	0	0	0	0	2.00	1.00	1.00
206.00	1	14	0	4.00	0	0	0	1	1	2	3	1	2	0	1.00	0	0	0	1	0	1	1	1	1	1	1	1	1	2.00	2.00	2.00
207.00	3	16	1	4.00	0	0	0	3	1	2	3	1	3	1	2.00	0	0	1	1	0	0	0	0	0	0	0	0	0	1.00	1.00	1.00
208.00	3	17	1	0.00	0	0	1	3	1	1	2	2	2	1	2.00	0	0	2	1	0	0	0	0	0	0	0	0	0	1.00	1.00	1.00
209.00	3	18	1	4.00	0	0	1	1	1	2	2	2	2	0	2.00	0	0	1	1	1	0	0	0	0	0	0	1	0	1.00	1.00	1.00
210.00	3	17	1	4.00	0	0	0	1	1	1	2	1	2	1	1.00	0	0	0	1	1	1	0	0	0	0	0	0	0	1.00	1.00	1.00
211.00	3	17	1	4.00	0	0	0	0	1	1	2	1	2	0	1.00	0	0	1	1	1	0	0	0	0	0	0	0	0	1.00	1.00	1.00
212.00	3	17	0	4.00	0	0	0	1	1	0	2	2	2	1	1.00	0	0	1	1	0	0	0	0	0	0	0	0	0	1.00	1.00	1.00
213.00	3	16	0	1.00	0	0	0	1	1	1	3	1	3	1	2.00	0	0	0	1	1	0	0	0	0	0	0	0	0	1.00	1.00	1.00
214.00	3	16	1	4.00	0	0	0	3	1	2	2	2	2	2	2.00	0	0	1	1	1	0	0	0	0	0	0	0	0	1.00	1.00	1.00
215.00	1	15	1	4.00	0	0	1	2	1	0	3	0	3	1	1.00	0	0	1	1	1	0	0	0	0	0	1	0	0	1.00	1.00	1.00
216.00	3	17	1	4.00	0	0	1	1	1	1	2	2	3	1	2.00	0	0	1	1	1	0	0	0	0	0	0	0	0	1.00	1.00	1.00
217.00	3	16	0	1.00	0	0	1	2	1	1	3	2	2	2	2.00	0	0	0	1	1	0	0	0	0	0	0	0	0	1.00	1.00	1.00
218.00	3	18	1	4.00	0	0	1	3	1	1	3	0	2	0	1.00	0	0	0	1	1	1	0	0	0	0	0	0	0	1.00	2.00	2.00
219.00	1	15	1	4.00	0	0	0	0	1	0	3	2	3	0	2.00	0	0	0	1	1	0	0	0	0	0	0	0	0	1.00	1.00	1.00
220.00	3	17	0	1.00	0	0	0	0	1	1	3	2	3	1	2.00	0	1	0	1	0	0	1	1	1	0	0	0	0	2.00	1.00	1.00
221.00	3	16	0	4.00	0	0	1	2	1	2	2	2	3	0	2.00	0	0	0	1	1	0	0	0	0	1	0	0	0	1.00	1.00	1.00
222.00	3	18	0	4.00	0	0	0	1	1	0	2	1	2	0	1.00	0	0	0	1	1	0	0	0	0	0	0	0	0	1.00	1.00	1.00
223.00	1	14	1	4.00	0	0	1	1	1	0	3	1	2	0	2.00	0	0	0	1	1	0	0	0	0	0	0	0	0	1.00	1.00	1.00
224.00	3	18	1	4.00	1	0	0	1	1	2	2	2	3	3	1.00	0	1	0	1	1	0	0	0	0	0	0	1	1	2.00	2.00	2.00
225.00	3	19	1	4.00	0	0	0	1	1	2	2	1	3	0	2.00	0	0	0	1	1	0	0	0	0	0	0	0	0	2.00	1.00	1.00
226.00	3	19	1	4.00	0	0	0	1	1	2	2	1	2	3	1.00	0	0	1	1	1	0	0	0	0	0	0	0	0	1.00	1.00	2.00
227.00	1	15	0	4.00	0	0	0	1	1	0	3	2	2	1	2.00	0	0	1	1	1	0	0	0	0	0	0	0	0	1.00	1.00	1.00
228.00	3	18	0	1.00	0	0	0	1	1	2	3	1	2	0	2.00	1	0	3	0	1	1	1	0	0	0	0	0	0	2.00	2.00	2.00
229.00	3	16	0	4.00	0	0	0	1	1	1	3	2	3	0	1.00	0	0	1	1	1	0	0	0	0	0	0	0	0	1.00	1.00	1.00
230.00	1	15	1	4.00	0	0	0	1	1	1	3	1	3	0	1.00	0	0	0	1	1	1	0	0	0	0	0	0	0	2.00	2.00	2.00
231.00	3	18	0	4.00	0	0	0	2	1	1	3	1	3	1	2.00	0	0	1	1	1	1	0	0	0	0	0	0	0	1.00	1.00	1.00
232.00	1	14	0	4.00	0	0	0	0	1	0	2	1	2	1	2.00	0	0	0	1	1	0	0	0	0	0	0	0	0	1.00	1.00	1.00
233.00	3	16	0	4.00	0	0	1	2	1	1	3	3	3	1	2.00	0	0	1	1	1	0	0	0	0	0	0	0	0	2.00	1.00	1.00
234.00	3	16	1	4.00	0	0	1	2	0	2	2	1	2	0	1.00	0	0	3	1	1	0	0	0	0	0	0	0	0	1.00	1.00	1.00
235.00	3	16	0	4.00	1	0	1	1	1	2	3	3	3	1	2.00	0	0	1	1	1	1	0	1	0	0	0	1	0	1.00	1.00	2.00
236.00	3	17	0	4.00	0	0	0	1	1	0	3	2	2	0	1.00	0	0	0	1	1	0	0	0	0	0	0	0	0	1.00	1.00	2.00
237.00	3	16	0	4.00	0	0	1	3	1	0	2	1	2	0	1.00	0	0	0	1	1	1	0	0	0	0	0	0	0	1.00	1.00	1.00
238.00	3	16	1	0.00	0	0	0	3	1	2	2	2	2	2	2.00	0	0	1	1	1	1	0	0	0	0	0	0	0	1.00	1.00	1.00
239.00	3	18	0	1.00	0	1	1	2	1	1	2	1	2	1	1.00	0	0	1	1	1	0	0	0	0	0	0	0	0	1.00	1.00	1.00
240.00	3	19	1	1.00	0	0	1	2	1	1	3	1	2	0	1.00	0	0	0	1	1	1	0	0	0	0	0	1	1	1.00	1.00	1.00
241.00	3	16	0	4.00	0	0	1	1	1	0	3	1	3	1	1.00	0	0	1	1	0	0	0	0	0	0	0	0	0	1.00	1.00	1.00
242.00	3	18	0	0.00	0	0	1	2	1	0	3	2	2	0	1.00	0	0	0	1	1	0	0	0	0	1	0	0	1	2.00	1.00	1.00
243.00	3	16	1	1.00	0	0	1	2	1	2	3	1	3	1	1.00	0	0	1	1	1	0	0	0	0	0	0	0	0	1.00	1.00	1.00
244.00	3	17	1	1.00	0	0	1	3	1	2	3	2	2	0	2.00	0	0	0	1	1	0	0	0	0	0	0	0	0	1.00	1.00	1.00
245.00	3	19	0	0.00	0	0	1	2	1	2	3	3	3	3	2.00	0	0	2	0	1	1	0	0	0	0	0	1	0	1.00	1.00	1.00

246.00	3	17	1	4.00	0	0	1	1	1	2	2	2	2	0	1.00	0	0	1	1	0	1	0	0	0	0	0	0	0	1.00	1.00	1.00
247.00	1	15	1	4.00	0	0	1	1	1	2	3	1	3	1	2.00	0	0	1	1	1	1	0	0	0	0	0	0	0	1.00	1.00	1.00
248.00	3	16	1	4.00	0	0	1	1	1	1	3	2	2	1	2.00	0	0	0	1	1	0	0	0	0	0	0	0	0	1.00	2.00	1.00
249.00	3	16	1	4.00	0	0	0	1	1	1	3	1	2	1	2.00	0	0	1	1	1	1	0	0	0	1	0	0	0	2.00	1.00	2.00
250.00	3	17	1	4.00	0	0	1	2	1	1	3	1	3	1	2.00	0	0	1	1	0	1	0	0	0	0	0	0	0	2.00	1.00	1.00
251.00	1	14	1	4.00	0	0	1	2	1	1	3	1	3	1	2.00	0	0	1	1	0	1	0	0	0	0	0	0	0	2.00	1.00	1.00
252.00	1	14	1	4.00	0	0	0	1	1	0	2	1	2	0	2.00	0	0	0	1	1	0	0	0	0	0	0	0	0	1.00	1.00	1.00
253.00	1	14	1	1.00	0	1	1	2	0	0	3	1	3	3	2.00	0	0	2	1	0	1	0	0	0	0	0	0	0	1.00	1.00	1.00
254.00	3	16	1	0.00	0	0	0	1	1	1	3	0	3	2	2.00	0	1	1	1	1	0	0	0	0	0	0	0	0	1.00	1.00	1.00
255.00	1	13	1	0.00	0	0	1	2	1	1	3	2	3	1	2.00	0	0	1	1	0	1	0	0	0	0	0	1	0	1.00	2.00	2.00
256.00	1	13	1	4.00	0	0	0	1	1	0	2	0	3	0	2.00	0	0	0	1	1	0	0	0	0	0	0	0	0	1.00	1.00	1.00
257.00	1	15	1	4.00	0	0	1	1	1	2	3	2	2	0	2.00	1	0	0	1	0	0	0	0	1	0	0	0	0	1.00	1.00	1.00
258.00	1	15	1	4.00	0	0	1	1	1	2	3	1	3	1	2.00	1	0	0	1	1	0	0	0	0	0	0	0	0	1.00	1.00	1.00
259.00	1	15	1	4.00	0	0	0	1	1	0	2	2	2	1	2.00	0	0	0	1	0	0	0	0	0	0	0	0	0	1.00	1.00	1.00
260.00	1	14	0	4.00	0	0	1	2	1	1	3	1	2	0	1.00	0	0	0	1	1	0	0	0	0	0	0	0	0	1.00	1.00	1.00
261.00	1	14	1	0.00	0	0	1	2	1	1	3	3	3	3	2.00	0	0	1	1	0	1	0	0	0	0	0	0	0	1.00	1.00	1.00
262.00	1	13	1	4.00	0	0	0	1	1	1	3	2	2	0	2.00	1	0	1	1	1	0	0	0	0	0	0	0	0	1.00	1.00	1.00
263.00	1	15	1	4.00	0	0	1	2	1	2	3	1	3	0	2.00	0	0	1	1	1	0	0	0	0	0	0	0	0	1.00	1.00	1.00
264.00	1	14	1	4.00	0	0	1	2	1	0	2	1	2	1	1.00	0	0	0	1	1	0	0	0	0	0	0	0	0	1.00	1.00	1.00
265.00	1	13	0	4.00	0	0	1	1	1	0	3	1	3	0	2.00	0	0	0	1	1	0	0	0	0	0	0	0	0	1.00	1.00	1.00
266.00	3	17	0	4.00	0	0	0	0	1	2	2	0	2	0	1.00	0	0	3	1	1	0	1	0	1	0	0	0	0	2.00	2.00	2.00
267.00	3	16	1	4.00	0	0	0	1	1	0	3	2	3	1	2.00	0	0	1	1	1	1	0	0	0	0	0	0	0	1.00	1.00	1.00
268.00	3	16	1	4.00	0	0	0	1	1	2	2	1	2	1	1.00	0	0	1	1	1	0	0	0	0	0	0	1	1	1.00	2.00	1.00
269.00	3	18	0	4.00	0	0	0	0	1	1	2	2	2	1	2.00	0	0	3	1	0	1	0	0	0	0	0	0	0	1.00	1.00	1.00
270.00	3	16	1	4.00	0	0	0	0	0	2	2	1	2	2	1.00	0	0	0	1	1	0	0	0	0	1	0	0	0	1.00	1.00	1.00
271.00	3	17	0	0.00	0	0	0	1	1	2	3	2	3	0	2.00	0	0	2	1	1	1	0	0	0	0	1	0	0	1.00	1.00	1.00
272.00	1	15	1	0.00	0	0	1	1	1	0	3	2	3	0	2.00	0	0	1	1	0	0	0	0	0	0	0	0	0	1.00	1.00	1.00
273.00	3	19	0	4.00	0	0	1	2	1	2	3	3	2	0	2.00	0	0	3	1	1	1	1	0	0	0	0	0	0	1.00	1.00	1.00
274.00	3	17	1	4.00	0	0	0	1	1	2	2	3	2	1	1.00	0	0	1	1	0	0	0	0	0	0	0	0	0	1.00	1.00	1.00
275.00	3	19	0	0.00	0	0	0	1	1	2	2	1	2	0	1.00	0	0	1	1	1	0	1	1	0	0	0	0	0	2.00	2.00	2.00
276.00	3	17	1	4.00	0	0	0	1	1	1	3	2	3	3	2.00	0	0	1	1	1	0	0	0	0	0	0	0	0	1.00	1.00	1.00
277.00	3	16	1	4.00	0	0	1	2	1	2	2	1	2	0	1.00	0	0	1	1	1	0	0	0	0	0	0	0	0	1.00	1.00	1.00
278.00	3	16	0	4.00	0	0	0	1	1	2	2	1	2	1	2.00	0	0	1	1	1	1	0	0	0	0	0	0	0	1.00	1.00	1.00
279.00	3	18	0	1.00	0	1	0	0	0	1	3	1	2	1	1.00	0	0	2	0	0	1	1	0	0	0	0	0	0	1.00	1.00	1.00
280.00	3	16	1	4.00	0	0	0	1	1	2	3	1	3	2	1.00	0	0	0	1	1	0	0	0	0	0	0	0	0	1.00	1.00	1.00
281.00	3	17	1	4.00	0	0	0	1	1	1	2	1	2	2	2.00	0	0	1	1	1	1	0	0	0	0	0	0	0	1.00	1.00	1.00
282.00	1	14	0	4.00	0	0	0	2	1	2	3	2	3	3	2.00	0	0	0	1	1	1	0	0	0	0	0	0	0	1.00	1.00	1.00
283.00	3	17	1	0.00	1	0	0	1	1	1	2	1	2	0	2.00	0	0	0	1	1	1	0	0	0	0	0	1	0	1.00	1.00	1.00
284.00	3	19	1	4.00	1	0	0	0	0	0	2	1	2	2	1.00	0	0	0	0	1	0	0	0	0	0	0	1	0	1.00	1.00	1.00
285.00	3	16	1	0.00	1	0	1	2	1	1	3	0	3	3	1.00	1	0	1	1	1	1	0	0	0	1	0	1	1	1.00	1.00	1.00
286.00	3	16	0	0.00	1	0	0	1	1	1	3	2	3	2	2.00	0	0	1	1	1	1	0	0	0	0	0	0	0	1.00	1.00	1.00
287.00	3	16	0	0.00	1	0	1	1	1	1	3	3	2	0	1.00	0	0	2	1	1	1	0	0	0	0	0	0	0	1.00	1.00	1.00
288.00	3	16	1	0.00	1	0	0	1	1	1	3	3	2	0	2.00	0	0	1	0	1	0	0	0	0	0	0	0	0	1.00	1.00	1.00
289.00	1	14	0	0.00	0	0	0	1	1	2	2	2	2	2	1.00	0	1	2	0	1	1	0	0	0	0	0	0	0	1.00	1.00	1.00
290.00	3	16	0	4.00	0	0	1	1	1	2	3	1	3	0	1.00	0	0	2	1	0	1	0	0	0	0	0	0	0	1.00	1.00	1.00
291.00	1	15	1	4.00	0	0	0	0	1	2	3	1	2	3	1.00	0	0	2	1	1	0	0	0	0	0	0	0	0	1.00	1.00	1.00
292.00	3	17	1	0.00	0	0	0	1	1	1	3	0	3	0	2.00	0	0	1	1	1	0	0	0	0	0	0	0	0	1.00	1.00	1.00
293.00	1	14	0	4.00	0	0	1	2	1	2	3	0	3	0	1.00	0	0	2	1	1	1	0	0	0	0	0	0	0	1.00	1.00	1.00
294.00	1	14	0	0.00	0	0	0	0	1	1	3	1	2	0	2.00	0	1	1	1	1	1	0	0	0	0	0	0	0	1.00	1.00	1.00
295.00	1	15	0	4.00	0	0	1	1	1	1	2	1	2	2	2.00	0	0	0	1	0	0	0	0	0	0	0	0	0	1.00	1.00	1.00
296.00	1	15	0	4.00	0	0	1	1	1	1	3	1	3	0	2.00	0	0	0	1	1	0	0	0	0	0	0	0	0	1.00	1.00	1.00
297.00	3	17	1	4.00	1	0	1	1	1	1	3	2	2	2	2.00	0	0	1	1	0	1	0	0	0	0	1	0	0	1.00	1.00	1.00
298.00	3	17	1	4.00	0	0	1	2	1	2	2	1	2	2	1.00	0	0	0	1	1	0	0	0	0	0	0	0	0	1.00	2.00	2.00
299.00	1	15	0	4.00	0	0	0	1	1	0	3	1	2	2	1.00	0	0	0	1	1	0	0	0	0	0	0	0	0	1.00	1.00	1.00
300.00	3	16	1	4.00	0	0	0	1	1	1	2	1	2	0	1.00	0	0	0	1	1	0	0	0	0	0	0	0	0	1.00	2.00	2.00
301.00	1	13	1	4.00	0	0	0	1	1	0	2	2	2	0	2.00	0	0	1	1	1	0	0	0	0	0	0	0	0	1.00	1.00	1.00
302.00	3	17	1	0.00	0	0	0	1	1	0	3	2	3	2	2.00	0	0	1	1	1	0	0	0	0	0	0	0	0	1.00	1.00	1.00
303.00	3	17	1	0.00	0	0	0	1	1	1	3	3	3	3	2.00	0	0	1	1	1	0	0	0	0	0	0	0	0	1.00	1.00	1.00
304.00	1	13	1	4.00	0	0	0	1	1	0	3	2	2	2	2.00	0	0	0	1	1	0	0	0	0	0	0	0	0	1.00	1.00	1.00
