# Supplementary material for: Prevalence and determinants of anxiety, depression, and suicidal ideation among adolescents of Parbat District: A cross-sectional study
Source: PLOS Ment Health. 2026 May 14;3(5):e0000613. doi: 10.1371/journal.pmen.0000613 (PMC13175331; doi:10.1371/journal.pmen.0000613)
Supplement: Attachment — Submitted filename: Response_to_Reviewers_FINAL.docx [file pmen.0000613.s002.docx]

**Author Response to Reviewer**

**“Prevalence and Determinants of Anxiety, Depression, and Suicidal Ideation among Adolescents of Parbat District: A Cross-Sectional Study” (PMEN-D-26-00096)**

**Revision – Round 1**

Dear Reviewer ,

We sincerely thank you for your valuable time and insightful comments on our manuscript titled **“Prevalence and Determinants of Anxiety, Depression, and Suicidal Ideation among Adolescents of Parbat District: A Cross-Sectional Study” (PMEN-D-26-00096)** submitted to PLOS Mental Health.

We appreciate the constructive feedback, which has helped us significantly improve the quality, clarity, and rigor of our manuscript. We have carefully addressed all comments and revised the manuscript accordingly. Below, we provide a detailed, point-by-point response to each reviewer comment. All changes have been clearly indicated in the revised manuscript with track changes and Highlights , and page/line numbers are provided for reference

| **Comments** | **Reviewer coment** | **Authors Response** | **Line No** |
| --- | --- | --- | --- |
|  | your Ethics Statement is available in its entirety at the beginning of your Methods section, under a subheading 'Ethics Statement'. | The ethical statement has been placed at the beginning of the Methods section under the subheading ‘Ethics Statement | Page 5  Line 113-125 ) |
|  | complete copy of PLOS’ questionnaire and data availability | questionnaire and data are attached as supplement file |  |
|  | separate figure files in .tif or .eps format. Also, remove the figures from your manuscript file but keep the legends. Please provide the source and license information for the base map layer. | Thank you for raising this important point. The map presented in Figure 1 was created using QGIS software. The base map shapefile used to generate the map was obtained from the Department of survey Government of Nepal(<https://dos.gov.np/> )The data provided by the Central Bureau of Statistics are publicly available for research and academic purposes. We confirm that the use of this data complies with the requirements of the Creative Commons Attribution (CC BY 4.0) license used by PLOS.We have now revised the figure legend to clearly indicate the source of the base map and its usage. | The figure has been updated and uploaded as a separate file (S1 Figure). |
| **Reviewer # 1** | | | |
| **R1.1** | This is a well-conducted cross-sectional study investigating the prevalence and associated factors of anxiety, depression, and suicidal ideation among high school adolescents in Parbat District, Nepal. The study addresses a critical public health gap in a rural, under-researched population. Methodologically sound with rigorous ethical standards, internationally validated instruments with excellent internal consistency (Cronbach's α > 0.92), and representative sampling across multiple schools, the paper provides valuable epidemiological data and identifies key risk factors. The findings are contextualized appropriately against national and global evidence. Overall, I recommend acceptance for publication with minor revisions to strengthen specific sections. | Response: We sincerely thank the reviewer for the positive and encouraging evaluation of our work. We appreciate the recognition of the methodological rigor and relevance of our study. We have carefully addressed all the suggestions to further strengthen the manuscript. |  |
| R1-2 | Abstract  The abstract is well-structured and comprehensively summarizes the study's background, methods, results, and conclusions. To enhance clarity and impact, I recommend that you emphasize the most striking finding in the results section: the extremely strong association between prior mental health history and suicidal ideation (aOR: 38.83; 95% CI: 4.71–320.26), which substantially exceeds the magnitude of other risk factors. Highlighting this would immediately draw readers' attention to the study's most clinically significant discovery. | Response: Thank you for this valuable suggestion. We agree that this is a key finding of high clinical relevance. We have revised the abstract to explicitly highlight the strong association between prior mental health history and suicidal ideation (aOR: 38.83; 95% CI: 4.71–320.26). | Change made: Revised in Abstract (Page 2 , Lines 35-50 ). |
| R1-3 | Introduction  The introduction effectively contextualizes the problem using both global and Nepal-specific epidemiological data. However, to strengthen the theoretical foundation, I recommend you expand the discussion of why women experience higher rates of anxiety versus depression. Beyond mentioning hormonal changes and exposure to sexual violence, consider briefly incorporating social-psychological mechanisms such as gender role socialization and differential stress appraisal patterns, as these may explain why the female odds ratio for depression (aOR: 5.65) is substantially higher than for anxiety (aOR: 2.59). This deeper mechanistic insight would provide better scaffolding for interpreting your results. | We appreciate this suggestion. We have expanded the Introduction to include social and psychological mechanisms such as gender role socialization, stress appraisal patterns, and coping styles to better explain the higher vulnerability of female adolescents to mental health problems. | Change made: Added in Introduction (Page 3 , Lines 80- 86). |
| R1-4 | The methodological approach is sound and well-documented. One clarification would strengthen the sample size calculation: explicitly state the source literature or prior study (reference 23 appears relevant) from which the baseline prevalence of 40.6% was derived, and briefly justify why this figure is appropriate for the Parbat population. Additionally, although the pretest was conducted in Phalewas Municipality while the main study occurred in Kushma Municipality (both within Parbat District), a brief note on whether the two municipalities differ substantially in socioeconomic or demographic characteristics would address potential concerns about whether the pretest findings generalize to the main study population. | Thank you for highlighting this important point. We have now explicitly cited the source study (Reference 23 ) from which the prevalence estimate (40.6%) was derived and justified its relevance to the study population. Additionally, we have included a clarification noting that Phalewas and Kushma municipalities share similar socio-demographic characteristics, supporting the applicability of the pretest findings. | Revised in Methods (Page 5 and 6, Lines 144 -161 ).  Pretest change ( page 6 , 176-188) |
| R1-5 | Results The results are presented clearly with well-organized tables and logically structured findings. However, one inconsistency deserves mention: the magnitude of the female gender effect differs substantially between anxiety (aOR: 2.59) and depression (aOR: 5.65). While both are significant, the paper would benefit from a brief explanatory statement in the results section noting this nearly 2.5-fold difference in risk ratios across the two conditions, preparing readers for the more detailed mechanistic discussion in the Discussion section. | Response: Thank you for this insightful observation. We agree that the substantially different magnitudes of the female gender effect across anxiety (aOR: 2.59; 95% CI: 1.30–5.19) and depression (aOR: 5.65; 95% CI: 1.82–17.53) represent an important and clinically meaningful finding. We have added an explanatory sentence in the Results section noting this nearly 2.5-fold difference in risk ratios across the two conditions. We acknowledge that while biological and social stressors elevate risk for both outcomes in female adolescents, the considerably stronger association with depression may reflect the differential roles of gender role socialization, ruminative coping styles, and perceived social expectations — factors elaborated further in the Discussion section. This addition prepares readers for the mechanistic interpretation provided later in the manuscript. | Added sentence in Results section (after non-significant factors paragraph for anxiety, before Table 3 heading)  ( page 12 and 13, line number 316-321 ) |
| R1-6 | Figures and Tables The tables are formatted appropriately and include necessary confidence intervals and p-values. I recommend adding a participant flow diagram (formatted according to STROBE guidelines) showing the number of students enrolled, those completing questionnaires, and any exclusions or missing data. Additionally, you might consider generating a forest plot displaying the adjusted odds ratios and their 95% confidence intervals for the three conditions side-by-side, which would allow readers to visually compare risk magnitudes across mental health outcomes and independent variables. | Response: We thank the reviewer for these constructive suggestions regarding visual presentation of the data. Regarding the participant flow diagram: our study used a school register-based systematic random sampling approach, and all 304 sampled students were present and consented to participate on the day of data collection, resulting in a 90% response rate with no exclusions due to missing data. We have added a brief note in the Methods section describing participant flow, including the total sampling frame (N=1,078), the calculated sample size (n=304), and the absence of exclusions, in lieu of a formal STROBE flow diagram, which is typically recommended for studies with staged enrollment or significant attrition. Regarding forest plots: we appreciate this valuable suggestion. We acknowledge that forest plots visually displaying the adjusted odds ratios and 95% CIs side-by-side across the three outcomes would enhance reader comprehension of risk magnitudes. We have noted this as a recommendation for future work; however, given the three separate multivariable models and the journal’s figure limits for the current submission, we have ensured the aOR tables are clearly formatted with 95% CIs and significance levels to allow ready comparison. | Participant flow note added to Methods. Tables retained with full CIs. Forest plot noted as future direction. |
| R1-7 | Discussion The discussion is comprehensive and appropriately contextualizes findings against existing literature. However, the interpretation of one finding—the protective effect of low school attendance (<20 days) against depression (aOR: 0.09; 95% CI: 0.01–0.82)—remains somewhat speculative. While you acknowledge the possibility of reverse causality (depressed adolescents missing more school), you do not sufficiently explore whether unmeasured confounders (e.g., students in alternative education settings, those working part-time) might explain this counterintuitive association. I recommend strengthening this section by explicitly acknowledging that this finding may reflect survival bias or population heterogeneity rather than a genuine protective mechanism of absenteeism. Furthermore, address whether sensitivity analyses using different school attendance cutoffs (e.g., <30 days) were performed to test the stability of this association. | Response: We are grateful for this important critique. We fully agree that the counterintuitive finding that school attendance fewer than 20 days was associated with reduced odds of depression (aOR: 0.09; 95% CI: 0.01–0.82) required a more rigorous and transparent discussion than was previously provided. We have substantially expanded this section in the revised manuscript to address all three dimensions raised by the reviewer: (1) Survival bias: We now explicitly acknowledge that only enrolled students who were present on the day of data collection could be included. Severely depressed adolescents who had already dropped out of school entirely would have been missed, creating a sampling bias whereby the low-attendance group may comprise a less severely affected subset of students. (2) Population heterogeneity and unmeasured confounding: We now discuss that students with very low attendance may include those engaged in vocational activities, part-time work, or alternative educational arrangements, who may face qualitatively different stressors than classroom-based peers, thereby confounding the attendance-depression relationship. (3) Sensitivity analyses: We acknowledge that sensitivity analyses using alternative attendance cutoffs (e.g., fewer than 30 days) were not feasible due to the small number of participants in the low-attendance category (n=14), which limits statistical precision as reflected in the wide confidence interval. We explicitly state this limitation and recommend that future studies with larger samples test the stability of this association across multiple thresholds to distinguish a genuine effect from artifacts of small cell size or sampling bias. These additions are clearly marked in the revised manuscript. | Expanded in Discussion section: survival bias, population heterogeneity, unmeasured confounders, and sensitivity analysis limitations all explicitly addressed.  Page 19 and 20  Line no 451-465 |
| R1-7 | Conclusions The conclusion accurately reflects the study's findings and appropriately identifies the urgent need for targeted mental health interventions. To enhance practical utility, I recommend that you be more specific about which groups require prioritized intervention: explicitly name female adolescents, non-Hindu religious minorities, students with documented childhood trauma, and those with prior mental health treatment history as distinct high-risk populations. Additionally, clarify the generalizability constraints: the study sample comprises only grades 11–12 (ages 16–19) within Kushma Municipality, and findings should not be extrapolated to early adolescents (ages 10–15), rural areas outside the district, or youth who have left formal schooling. | We appreciate this valuable suggestion. We have revised the conclusion to explicitly identify high-risk groups, including female adolescents, non-Hindu religious minorities, those with a history of childhood trauma, and individuals with prior mental health conditions. We have also clarified the limitations in generalizability, noting that the findings are specific to school-going adolescents (grades 11–12) in Kushma Municipality and may not be applicable to younger adolescents or out-of-school populations. | Page 22  Line( 543-553) |
|  | Minor comments |  |  |
| **Reviewer #2:** | | | |
| R2-1 | 1. Sampling Procedure and Representativeness Although the study mentions systematic and proportionate sampling across schools, more detail is needed regarding how schools and participants were selected. The authors should clarify whether the sampling approach ensures representativeness of adolescents in the district. Additionally, the inclusion of only school-going adolescents may limit the generalizability of the findings to adolescents who are not enrolled in school. |  | Revised in Methods (Page 5 and 6, Lines 144 -161 ). |
| R2-2 | 2. Sample Size Justification The manuscript would benefit from a clearer explanation of the sample size calculation, including the assumptions used (expected prevalence, confidence level, margin of error). This information is important for evaluating whether the sample size is adequate to detect meaningful associations. |  | Revised in Methods (Page 5 and 6, Lines 144 -161 ). |
| R2-3 | 3. Logistic Regression Modeling The authors report using multivariable logistic regression models to identify factors associated with anxiety, depression, and suicidal ideation. However, additional details regarding the model-building process would strengthen the methodological transparency. Specifically, the authors should clarify: Whether multicollinearity among independent variables was assessed Whether model goodness-of-fit statistics were evaluated The criteria used to select variables for the final models. | Response: Thank you for this important request for methodological transparency regarding the model-building process. We have now added a dedicated paragraph to the Statistical Analysis section of the Methods explicitly addressing all three points raised: (1) Multicollinearity assessment: Multicollinearity among independent variables was assessed using the Variance Inflation Factor (VIF). All VIF values were below 5, indicating acceptable collinearity among predictors and confirming the stability of the multivariable models. (2) Model goodness-of-fit: Model fit was evaluated using the Hosmer-Lemeshow test for each of the three multivariable logistic regression models. All three models demonstrated adequate goodness-of-fit (p > 0.05), indicating that the models fitted the data satisfactorily. (3) Variable selection criteria: Variable selection followed a pre-specified two-stage approach. First, all candidate variables were screened at the bivariate level using chi-square tests; those with p-value ≤ 0.25 were eligible for inclusion in the multivariable model. In the second stage, selected variables were entered simultaneously into the multivariable logistic regression model, and final inclusion was determined by statistical significance at p < 0.05, adjusted for other covariates, alongside clinical and theoretical relevance. These clarifications have been incorporated into the revised manuscript with tracked changes. | Added in Statistical Analysis section of Methods: VIF multicollinearity check, Hosmer-Lemeshow goodness-of-fit, and two-stage variable selection criteria.  Page no 8 line no 261-269 |
| R2-4 | 4. Counterintuitive Association with School Attendance The analysis suggests that poor school attendance is associated with lower odds of depression. This finding appears counterintuitive and may reflect issues related to variable coding, confounding, or measurement. The authors should clarify how school attendance was categorized and discuss potential explanations for this result. | Response: We thank the reviewer for flagging this important methodological concern. Regarding variable coding: school attendance was categorized as a binary variable — fewer than 20 days vs. 20 days or more per month — based on the school attendance register, and this categorization was pre-specified before analysis. The coding direction was confirmed: students attending fewer than 20 days served as the reference category in the model, and the aOR of 0.09 indicates that students attending 20 or more days had 91% lower odds of depression compared to those with fewer than 20 days of attendance, a finding which appears counterintuitive. We have now substantially expanded the Discussion to address three plausible explanations: (a) Survival bias — only enrolled, attending students could be sampled; severely depressed students who dropped out were not captured; (b) Unmeasured confounding — low-attendance students may include those engaged in vocational or part-time work and experiencing different kinds of stressors not captured in the questionnaire; (c) Reverse causality — depression itself may cause absenteeism, and the cross-sectional design cannot determine directionality. The very small cell size (n=14 in the low-attendance group) and correspondingly wide confidence interval (0.01–0.82) further caution against strong conclusions. These limitations are now explicitly acknowledged in the revised Discussion with tracked changes. | Coding confirmed in Results. Expanded in Discussion with survival bias, unmeasured confounding, and reverse causality explanations. Small subgroup limitation noted. |
| R2-4 | 5. Figures The results section would benefit from the inclusion of graphical visualizations (e.g., bar charts for prevalence estimates or forest plots for adjusted odds ratios). These figures would improve the readability and interpretation of the findings. | Response: We thank the reviewer for this constructive recommendation. We appreciate that bar charts for prevalence estimates and forest plots for adjusted odds ratios would substantially improve the visual accessibility and interpretability of our findings. Regarding prevalence bar charts: the prevalence of the three outcomes (anxiety 36.5%, depression 17.4%, suicidal ideation 7.6%) is concisely presented in Table 2 of the manuscript. Given journal figure limits, we have retained this tabular format but have ensured the Results narrative clearly summarizes and contrasts these figures. Regarding forest plots for adjusted odds ratios: we agree this would be a valuable addition. The three separate multivariable logistic regression tables (Tables 3, 4, and 5) with aORs and 95% CIs are formatted to allow direct visual comparison across outcomes. We acknowledge this as a limitation of the current submission format and commit to including a combined forest plot as a supplementary figure if permitted by the journal editor, or as a priority visual in any revised or extended version of this work. The reviewer’s suggestion has been noted in the revised manuscript. | Tables retained with full CIs. Forest plot committed as supplementary figure if permitted. Acknowledged in manuscript. |
| R2-5 | Minor Comments: | We have carefully revised the manuscript for language clarity, reduced redundancy in the background, ensured consistent terminology, and improved table presentation by including both counts and percentages. |  |

We sincerely thank the editor and reviewers once again for their valuable feedback. We believe the revisions have significantly strengthened the manuscript and addressed all reviewer concerns. We hope it is now suitable for publication in PLOS Mental Health.

Sincerely,
Ms. Anjali P.C
(On behalf of all authors)
